# Engagement of National Stakeholders and Communities on Health-Care Quality Improvement: Experience from the Implementation of the Partnership for HIV-Free Survival in Tanzania

**DOI:** 10.1177/2325958219847454

**Published:** 2019-06-13

**Authors:** Stella Kasindi Mwita, Monica M. Ngonyani, Jane Mvungi, Roland A.M. van de Ven, Theopista Jacob Masenge, Davis Rumisha, Deborah Kajoka, Grace Dennis, Aurora O. Amoah

**Affiliations:** 1 US Agency for International Development (USAID) Applying Science to Strengthen and Improve Systems (ASSIST) Project, University Research Co, LLC (URC), Tanzania, United Republic of Tanzania; 2 Elizabeth Glaser Pediatric AIDS Foundation, Washington, DC, USA; 3 Baylor College of Medicine Children’s Foundation, Mbeya, Tanzania, United Republic of Tanzania; 4 Ministry of Health, Community Development, Gender, Elderly and Children, Tanzania, United Republic of Tanzania; 5 Research and Evaluation Consultant, USAID ASSIST Project, URC, MD, USA; 6 Data Analytics Research and Evaluation Group, Washington, DC, USA

**Keywords:** HIV, PMTCT, nutrition, quality improvement, community

## Abstract

The Partnership for HIV-Free Survival initiative in Tanzania integrated postnatal nutrition and mother-to-child transmission (MTCT) cascades to reduce vertical HIV transmission. Quality improvement (QI) was implemented in 30 health facilities. Net positive gain resulted in overall improvement in all indicators (above 80%) by the end of the reporting period. Retention in postnatal care (mean = 49.8, standard deviation [SD] = 27.6) and in monthly HIV services (mean = 65.4, SD = 29.5) had the lowest average but showed consecutive and significant (*P* ≤ .001) gains except for significant decreases in 1 of 6 periods assessed. Average antiretroviral therapy uptake among women (mean = 81.7, SD = 29.5) was highest, with an initial positive gain of 78.9% (*P* ≤ .001). DNA/polymerase chain reaction for HIV-exposed infants (mean = 71.8, SD = 20.9) and nutrition counseling (mean = 71.2, SD = 26.3) showed similar average performance, with the latter being the only indicator with significant equal periods of gain and decreases. The collaborative QI approach improved process indicators for reducing MTCT in resource-constrained health systems.

**What Do We Already Know about This Topic?**

We know that vertical transmission specifically through delivery and breast-feeding is the main mode that needs to be addressed if we are to eliminate MTCT to less than 5%.

**How Does This Research Contribute to the Field?**

We tested and documented implementation solutions for addressing PMTCT challenges which helped HIV+ pregnant and lactating women.

**What Are the Implications for Theory, Practice, or Policy?**

This initiative led to policy changes that are being spread to other health facilities in Tanzania to reduce transmission of HIV to infants.

## Introduction

HIV transmission risk from mother to child is heightened during pregnancy, childbirth, and breast-feeding.^1^ Although research has identified cost-effective best practices for prevention of mother-to-child transmission (PMTCT),^2^ vertical transmission remains a highly problematic aspect of the HIV/AIDS epidemic in sub-Saharan Africa, where 90% of all HIV-positive children live. In eastern and southern Africa, new infections fell by only 12.7% in 5 years, from 1.1 million to 960 000 between 2010 and 2015.

Tanzania adopted the World Health Organization’s (WHO) PMTCT strategy by focusing on 4 ways to eliminate vertical transmission: (1) reducing HIV incidence among women; (2) increasing access to family planning among women; (3) reducing vertical transmission to less than 5%; and (4) reducing maternal and child mortality by 90% by 2015.^3^ The for prevention of mother-to-child transmission services have been available in Tanzania since 2000. Tanzania attained 93% integration of PMTCT with reproductive and child health (RCH) services, and 85% of pregnant women were tested for HIV during antenatal care clinic (ANC) visits. By 2013, 77% of HIV-positive pregnant women were receiving antiretroviral (ARV) prophylaxis.^3^

However, vertical transmission remained unacceptably high at 18%. There was a 42.3% reduction in MTCT between 2009 (26 000 children infected with HIV) and 2012 (15 000 children infected with HIV) and a 55.4% increase in 2013 (26 000 children), despite the fact that the country reported a 25% increase in treatment coverage between 2010 and 2015.^3,4^ The inability to curb vertical transmission has been attributed to a combination of limited access to care and poor service delivery in a resource-constrained health system.^5^ At the program level, there has been difficulty retaining mothers and their HIV-exposed infants (HEI) in care, providing patients with the recommended ARV drugs and addressing the nutritional status of both mothers and infants.^6,7^

The Partnership for HIV-Free Survival (PHFS)^8^ was a major initiative targeted to 6 countries (Kenya, Lesotho, Mozambique, South Africa, Tanzania, and Uganda) with high HIV prevalence in eastern and southern Africa. The country-led initiative was designed to assist national efforts to improve postnatal, maternal, and infant HIV care and nutrition support through effective implementation of the 2010 WHO Guidelines on HIV and Infant Feeding. In Tanzania, PHFS started in October 2013 with the goal of reducing MTCT and improving the survival of mothers and infants. The PHFS goals were integrated with existing PMTCT structures and programs and aligned with Tanzania’s HIV treatment policy shift from option A to option B+. Option B+ was added to option A, which was the WHO-recommended daily ARV prophylaxis for infants from birth to 6 weeks of age, regardless of feeding method. Option B+ added lifelong ART therapy for HIV-positive pregnant women from point of diagnosis regardless of CD4 count.^9,10^ Through PHFS, Tanzania focused on improving enrollment and retention on ART for HIV-positive pregnant women and lactating mothers, strengthening access to early infant HIV diagnosis and improving safe infant and young child feeding (IYCF) practices, including exclusive breast-feeding for the baby in the first 6 months.

A quality improvement (QI) approach was employed by PHFS in all 6 countries to identify gaps in health services (described in the JIAPAC-18-08-SA-1098 paper in this supplement). Quality improvement has proven effective in strengthening service quality for HIV-positive mothers and infants in health systems with limited resources, in conjunction with other factors including strong leadership, collaboration, and resource allocation.^5,11^ This article describes a collaborative QI approach applied in 3 regions in Tanzania (Mbeya, Iringa, and Tabora) from October 2013 to December 2015.

## Ethical Consideration

The data are deidentified administrative data that were aggregated by facility and then by district. This work was exempt from the institutional review board approval process.

## Methods

The QI approach was implemented within routine PMTCT programs^10^ in the selected health facilities. Quality improvement is a management science that equips front-line health workers with skills and tools to change facility-level systems and processes of care (described in the JIAPAC-18-08-SA-1100 paper in this supplement). Quality improvement strategies were applied to improve integration of services and retention in care for mothers and babies in the program, with the goal of ensuring their optimal health including by keeping exposed infants HIV free. Using plan-do-study-act (PDSA) cycles,^12^ facility QI teams tested small, measurable changes to effect improvement in clinic processes. The PDSA approach is an iterative process of identifying gaps, planning activities (change ideas) to address those gaps, implementing and studying the impact of those changes, and then deciding whether to adopt, adapt, or discard changes based on whether or not they resulted in improvement.

### QI Partnership and Coordination under the National Steering Committee

**Figure 1.**
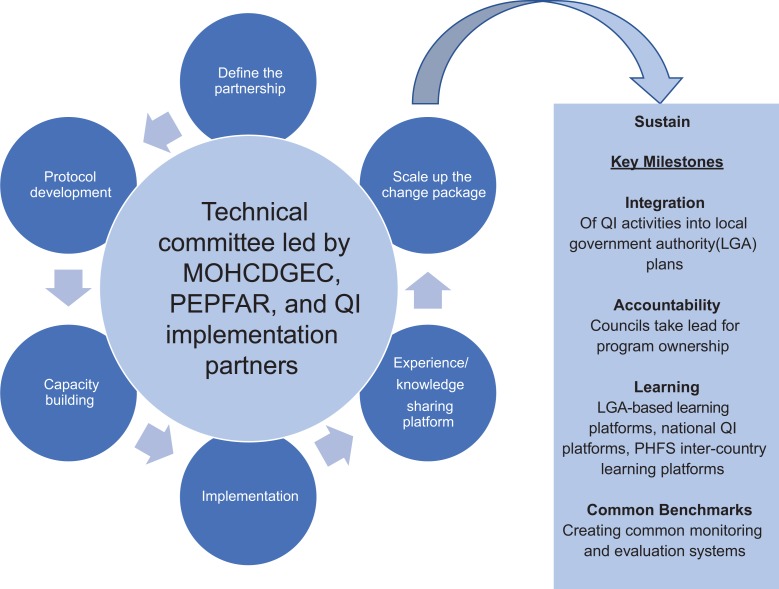
PHFS implementation process in Tanzania. PHFS indicates Partnership for HIV-Free Survival.

Figure 1 describes the coordination of PHFS in Tanzania. Six primary areas of coordination (define the partnership, protocol development, capacity building, implementation, sharing experience and knowledge, and scale up using the change package derived from the QI experience) were helmed by the National Steering Committee (NSC). The NSC was formed by the PMTCT unit of Tanzania’s Ministry of Health, Community Development, Gender and Children (MOHCDGEC) to provide guidance and oversight throughout the PHFS implementation period. Technical support and capacity building for QI were provided to the NSC, implementing partners, and facility QI teams by the US Agency for International Development (USAID) Applying Science to Strengthen and Improve Systems (ASSIST) project.

Defining the partnership was the critical first step, as PHFS activities within the facilities were led by different partner organizations in each of the 3 regions: Elizabeth Glaser Pediatric AIDS Foundation in Nzega District of Tabora Region; Deloitte–Tunajali—a Tanzania-based nongovernmental organization—in Mufindi District of Iringa Region; and Baylor International Pediatric AIDS Initiative, supporting the Mbeya Municipal Council in Mbeya Region. The NSC developed a national protocol for PHFS implementation, which clearly stipulated each stakeholder’s roles and responsibilities. Throughout PHFS implementation, the committee met monthly to share results, insights, and challenges.

### Selection of the Demonstration Sites

**Figure 2.**
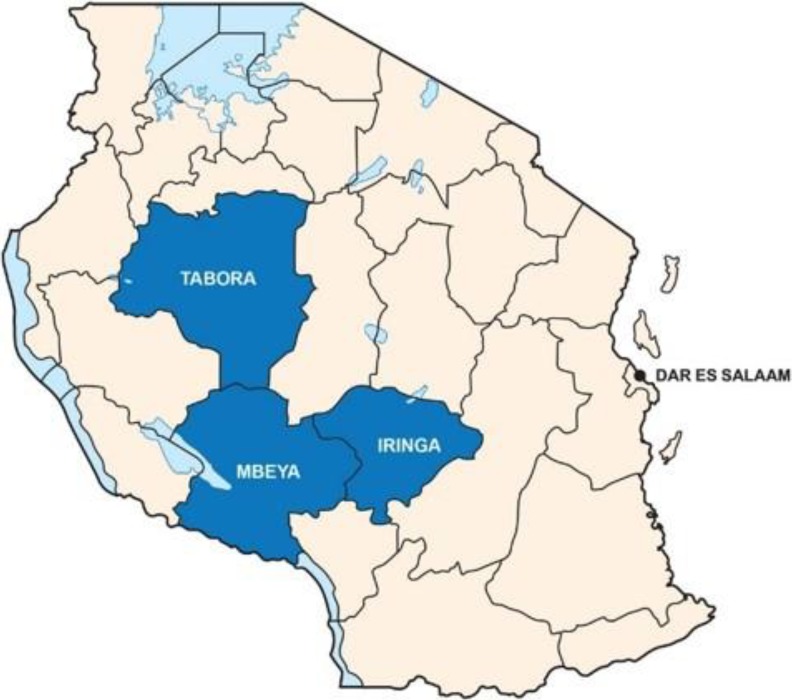
PHFS regions in Tanzania. PHFS indicates Partnership for HIV-Free Survival.

There is geographic variability in HIV prevalence in Tanzania.^4^ Figure 2 shows the selected PHFS implementation regions on a map of Tanzania. These regions have a high HIV prevalence, particularly Mbeya and Iringa at 14% and 13%, respectively. Both rates were above that of the primary urban area, Dar es Salaam (11%), in 2002.^13^ Although the prevalence had dropped by 2012, they were still considered high HIV-prevalence regions with Iringa at 9.1%, Mbeya at 9%, and Tabora at 5.1%.^14^ Within the 3 regions, the selected districts have a high HIV prevalence: 13.8% in Mufindi (Iringa region), with 19 373 people living with HIV (PLHIV); 9.85% in Mbeya (Mbeya region), with 28 928 PLHIV; and 4.5% in Nzega (Tabora region), with 14 168 PLHIV.^7^ These districts also had low rates of retention in care among HIV-positive pregnant and lactating mothers. Thirty high-volume health facilities (10 in each district) were selected as PHFS demonstration sites.

### QI Process

**Figure 3.**
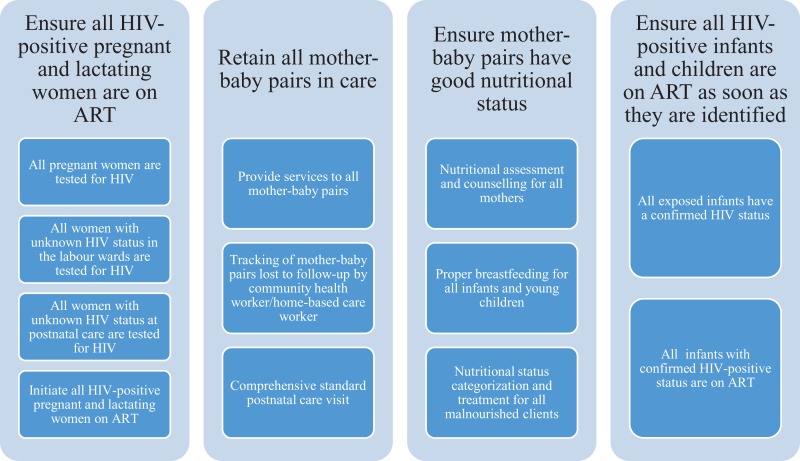
PHFS QI framework and indicators. PHFS indicates Partnership for HIV-Free Survival; QI, quality improvement.

The NSC initially identified the following 4 key care areas of relevance to the PHFS objectives (Figure 3): (1) ensure that all HIV-positive pregnant women and lactating mothers were on ART; (2) improve retention in care for mothers and their babies; (3) ensure good nutrition status for mothers and babies; and (4) ensure that all HIV-positive infants were started on ART as soon as they were identified. The ASSIST, in collaboration with the 3 implementing partners, identified a package of changes to guide teams in improving the 4 care areas, using 10 indicators to monitor results.

### Work of QI Teams and Support to Teams

Quality improvement teams were formed in the 30 facilities; teams comprised of a diverse cadre of health workers including clinicians, nurses, and allied health professionals. The ASSIST staff, in collaboration with the regional partners, trained the teams. The QI teams were coached by district coaching teams to implement and document the QI process: identifying gaps deciding on what changes to make to clinic processes, testing those changes using PDSA cycles, measuring impact by monitoring relevant indicators using standard evaluation system (SES) journals,^15^ and deciding if a given change resulted in improvement.

### Collaboration and Shared Learning across Teams

Each QI team took part in 4 collaborative learning sessions spread across the implementation period. To maximize learning, sessions were convened for 3 to 4 teams at a time and included QI process refreshers to ensure participants returned to their facilities well-equipped to share and apply what they learned. Regional and Council Health Management Teams (R/CHMTs) participated in the learnings sessions too and offered mentorship to QI teams between the sessions.

The NSC also coordinated national-level sharing sessions for all 3 districts. At these sessions, key learnings (such as high-improvement change ideas) were disseminated to all 30 sites (and later to additional sites on those districts which were chosen to scale up the PHFS work). The key learnings were documented and compiled into a change package for Tanzania.^16^

### Community Involvement

Volunteer community members facilitated mother–baby pairs’ linkage to the standard package of postnatal health-care services promoted under the PHFS initiative. Community volunteers were already supporting home-based care activities for HIV-positive clients. Their main role was supporting pregnant and lactating women and their babies to attend and remain in care. Key operational activities started with community-level household mapping for all pregnant women and lactating mothers. Each community focal person was assigned specific households for periodic follow-up to encourage attendance at antenatal care at health facilities. Regular (usually monthly) meetings between an assigned facility supervisor and community volunteers facilitated community-facility linkages and continuous feedback. Facility supervisors were usually a health worker who was part of the QI team at that health facility.

### Measuring Improvement

Indicators to measure improvement were selected from the following areas: HIV testing, retention, ART, and nutrition. These indicators were selected to monitor the continuum of care of integrated PMTCT services. Table 1 shows the main changes tested in each of these areas, corresponding indicators, and the data sources used by teams to measure their effectiveness. Data to calculate the indicators were extracted monthly from 4 clinic registers by QI team members. These registers were the ART Patient Register, PMTCT Mother-Child Follow-up Register (MCFU), used to track HEI from birth to 18 months; the postnatal care register, used to track mothers from delivery to 42 days after delivery; and Nutrition Assessment, Counselling, and Support (NACS) registers, which document nutritional assessment and categorization. The ASSIST and partners verified the SES journals while helping the teams to improve tracking, documentation, and data collection. Health workers at the facilities also used Care and Treatment Center (CTC2) and HEI cards to document individual progress through the ART and PMTCT programs. The CTC2 database is a national-level database created by the National AIDS Control Program that collects patient-level data at the site level and tracks different areas related to HIV, such as ART and tuberculosis. Progress on the indicators was captured in databases and in QI journals, which the QI teams compiled in a QI-specific, site-level file. This review of indicators’ progress was usually done monthly.

**Table 1.** Changes Tested by QI Teams by Area of Improvement.

Area of Improvement	Changes Tested	Indicator	Numerator	Data Source	Denominator	Data Source
Retention of mothers and babies	Appointments given to home-based care (HBC) workers for tracking. Writing on the mother’s card the specific date and day that she should come to the clinic	Percentage of all mother attended all postnatal care (PNC) standard visit (2, 7, 28, and 42 days)	Number of all mothers attending all PNC standard visits	MTUHA # 13 Row 2a	All postnatal mother eligible to attend all standard PNC visits (all women delivered in the past 6 weeks)	MTUHA 13 Row 9 Columns 11 and 12
	Giving same-day appointments for the mother and infant to come for services—same date and place. Stapling together mother’s Care and Treatment Center (CTC2) cards to HEI cards, ensuring mothers and babies will be seen together.	Percentage of HIV-positive mothers and babies attending HIV service each month	Number of HIV-positive mothers and babies attending HIV services each month	ART register or database	Total number of HIV+ mothers and babies eligible to attend CTC/RCH	ART register or database
ART for HIV-positive pregnant women, postnatal women and infants/children <2 years	Recording contacts of the mothers whose babies have polymerase chain reaction (PCR) results and making phone calls to track them. Improved documentation in the Mother-Child Follow-Up (MCFU) register. One focal person allocated to track PCR results at the facility	Percentage of HIV-positive pregnant women currently on ART	Number of HIV-positive pregnant women currently on ART	CTC2 Database, ART Register	Total number of HIV-positive pregnant women during the reporting period	MTUHA 6, ART Register
	Timely ordering of adequate stock of ARV and re-agents. Request ARV from other facilities in times of shortages. Counseling mothers on the importance of taking ARV and dangers of not taking them	Percentage of exposed infants tested for HIV through PCR and received results	Number of exposed infants tested through PCR and received results	MCFU, Summary forms, MTUHA 8 & 13	Number of exposed infants registered tested	MCFU, Summary forms, MTUHA 8 & 13
Nutritional assessments & counseling	Assign specific points/health-care workers at RCH for nutritional assessments and counseling. Link community-based nutritional support initiatives to the health facility through referral	Percentage of HIV-positive pregnant women and postnatal women who attend RCH and are counseled on nutrition	Number of HIV-positive pregnant women and postnatal women who attend RCH and are counseled on nutrition	Nutrition assessment counseling and support (NACS) Register	Total number of HIV-positive pregnant and postnatal women who attend RCH during that month	NACS Register, PNC Register, ART Register

Abbreviations: ARV, antiretroviral; HEI, HIV-exposed infants; RCH, reproductive and child health.

### Analysis of Improvement Trends

The 5 indicators selected for analysis present a picture of progress across the QI framework. We charted the trend of the selected indicators over a 3-year period from June 2013 to June 2016. Trends for each of the 3 districts were compared to the aggregate trend across all sites. The overall median line for each indicator was included to assess the sustainability of the improvement over time. The 3-year duration of the initiative was divided into 6 periods. For each of the 5 indicators, we used a Pearson χ^2^ test to determine the statistical significance of the improvement from the start of every period to the endpoint of every period. The χ^2^ assessed the association between patients receiving the intervention before and after 6-month intervals over the 3-year period from June 2013 to June 2016.

## Results

### Partnership for HIV-Free Survival Change Package

The implementation of PHFS resulted in a change package that combined best practices across demonstration sites.^16^ All the 30 health facilities used the same indicators and the same tested changes. Results were shared in the 3 national-level PHFS sharing meetings. At the second meeting, health workers shared successes and challenges with regard to the changes. From this meeting, 4 or 5 changes per indicator were selected and packaged for dissemination by the MOHCGGEC that was later used to support QI activities at 60 other facilities in the same districts after the initial PHFS implementation.

Table 1 shows a summary of the key changes associated with the selected indicators. The tested changes pertaining to retaining mothers and their babies included keeping their records together, giving same-day appointments, and properly documenting the date and time of the follow-up appointment for the mothers.

To facilitate ART uptake among HIV-positive women, facilities emphasized the timely ordering of ARV drugs and reagents or requested them from neighboring facilities in the event of a shortage. Mothers were also counseled about the importance of adhering to the treatment regimen.

HIV-exposed infants testing and delivery of results are important in identifying infants who must be started on ART. Improving record-keeping, tracking test results, and making sure the facility recorded contact information for the mothers/caregivers are key factors. Proper documentation and drug management including the ability to quickly have ARV drugs on hand and in the right dose for infants were critical. To facilitate nutrition counseling, health workers were assigned to provide nutritional counseling at specific points in the RCH clinic, and a referral system was developed to link target clients to community-based nutritional programs.

#### Retention of mother–baby pairs

**Figure 4.**
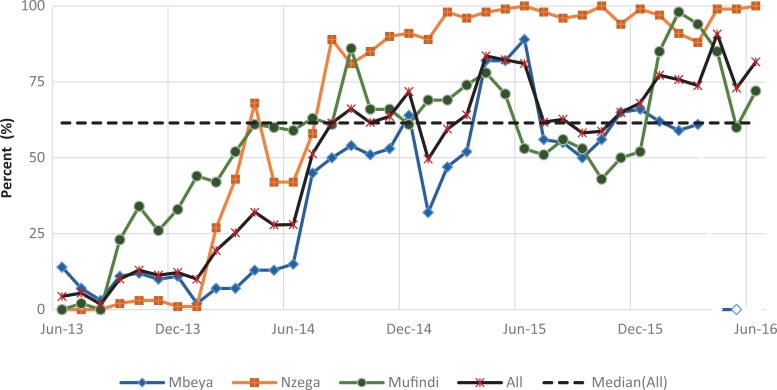
Mothers attending all 4 PNC visits at 2, 7, 28, and 42 days (June 2013 to June 2016). PNC indicates postnatal care.

**Figure 5.**
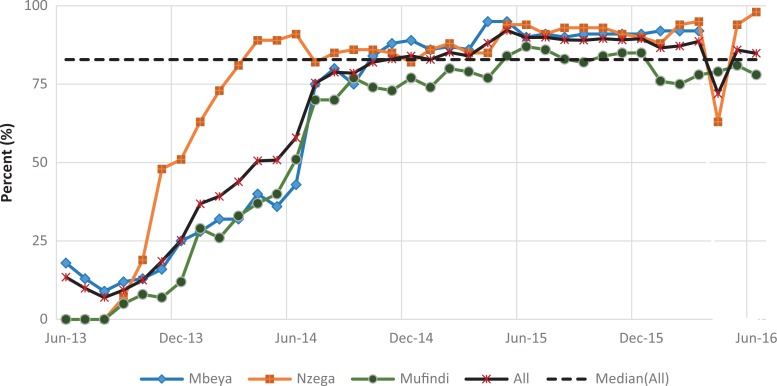
Figure HIV-positive mothers and babies attending HIV services each month (June 2013 to June 2016). PNC indicates

Figure 4 shows the trend for the percentage of mothers attending the 4 recommended PNC visits (indicator 1), while Figure 5 shows the percentage of HIV-positive mothers and infants attending HIV services each month (indicator 2). Both indicators were very low for all districts at baseline but increased quickly in the first year. The mean for indicator 1 was 49.9 (standard deviation [SD] = 27.6), with a low of 1.7%, and then reached a maximum of 90.8% (Table 2). The indicator attained some level of improvement when it was able to move above the overall median of 61.5% after a year of implementation in August 2014. The 6-month change in the indicator was highly significant in each period. In period 1, there was an increase of 7.9% over the low baseline of 4.4% (*P* ≤ .001), a gain of 15.8% (*P* ≤ .001) in period 2, 43.8% (*P* ≤ .001) in period 3, and a relatively small gain of 9.2% (*P* ≤ .001) in period 4. By period 5, the indicator decreased significantly by 13.1% (*P* ≤ .001) after reaching 81%, but it increased again by 13.7% to a slightly higher value of 81.6% by end of the reporting period (*P* ≤ .001; Table 3).

**Table 2.** Summary Measures for the 5 Indicators, June 2013 to June 2016.

Indicator	Definition	Mean	Median	Standard Deviation	Minimum	Maximum
**1**	**Percentage of mothers attending all 4 postnatal care (PNC) standard visits (2, 7, 28, and 42 days)**	**49.8**	**61.5**	**27.6**	**1.7**	**90.8**
N	Number of all mothers who attended all PNC standard visits	503	614	304	4	928
D	All postnatal mothers eligible to attend all standard PNC visits (all women delivered past 6 weeks)	955	1058	265	231	1282
	Number of facilities reporting	27	30	5	14	30
**2**	**Percentage of HIV-positive mother–baby pairs attending HIV services each month**	**65.4**	**82.9**	**29.5**	**7.0**	**92.1**
N	Number of HIV-positive mothers and babies attending HIV services each month	1703	1665	1131	55	3252
D	Total number of HIV-positive lactating mothers enrolled in the program (all lactating mothers who are in ART program)	2240	2119	963	785	3741
	Number of facilities reporting	27	30	5	15	30
**3**	**Percentage of HIV-positive pregnant and lactating women on ART in the month**	**81.7**	**87.9**	**20.9**	**10.9**	**96.0**
N	Number of HIV-positive pregnant and lactating women actively on ART	2532	3011	1627	25	4603
D	Total number of HIV-positive pregnant and lactating women during the reporting period (all HIV-positive pregnant and lactating women who are supposed to get ARV in that month) (cumulative)	2899	3481	1847	81	5322
	Number of facilities reporting	27	30	5	12	30
**4**	**Percentage of exposed infants tested for HIV through PCR and receive results**	**71.8**	**77.1**	**19.7**	**20.2**	**96.9**
N	Number of results given to parents/guardians within 4 weeks after they reached the facility	119	122	59	11	219
D	Total number of results received at the facility in the reporting month	157	164	63	46	271
	Number of facilities reporting	26	29	6	13	30
**5**	**Percentage of HIV-positive pregnant women and postnatal women who attend RCH and are counseled on nutrition**	**71.2**	**79.1**	**26.3**	**7.8**	**98.5**
N	Number of HIV-positive pregnant women and postnatal women who attend RCH and are counseled on nutrition	1936	1873	1415	16	4490
D	Total number of HIV-positive pregnant and postnatal women who are assessed for nutrition during the reporting month	2339	2247	1429	62	4574
	Number of facilities reporting	25	28	7	9	30

Abbreviations: ART, antiretroviral therapy; ARV, antiretroviral; PCR, polymerase chain reaction; RCH, reproductive and child health.

**Table 3.** Assessing Statistical Significance of Improvement on Indicators over 6-Month Intervals (June 2013 to June 2016).^a,b^

Indicator	Periods-		1	2	3	4	5	6	
	Definition	June 13	December 13	June 14	December 14	June 15	December 15	June 16	Charts
**1**	**Percentage of mothers attending all 4 postnatal care (PNC) standard visits (2, 7, 28, 42 days)**	**4.4**	**12.2**	**28.0**	**71.8**	**81.0**	**68.0**	**81.6**	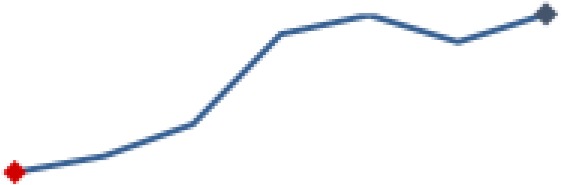
N	Number of all mothers who attended all PNC standard visits	31	139	329	510	915	853	489	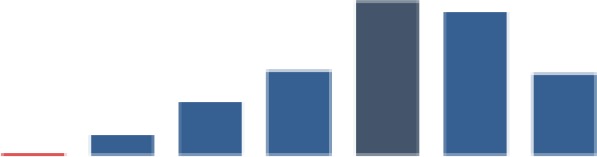
D	All postnatal mothers eligible to attend all standard PNC visits (all women delivered past 6 weeks)	710	1136	1175	710	1129	1255	599	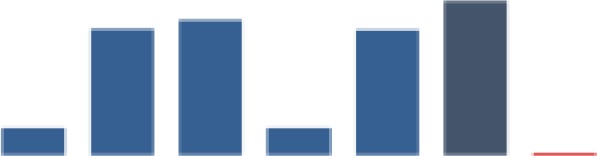
	6-month change in indicator		7.9	15.8	43.8	9.2	(13.1)	13.7	
	χ^2^ test		^e^	^e^	^e^	^e^	^e^	^e^	
	Number of facilities reporting	19	29	30	28	30	30	19	
									
**2**	**Percentage of HIV-positive mother-baby pairs attending HIV services each month**	**13.5**	**25.2**	**58.0**	**84.0**	**89.9**	**89.5**	**84.9**	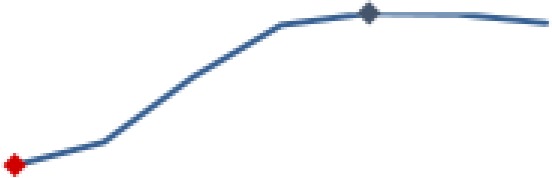
N	Number of HIV-positive mothers and babies attending HIV services each month	108	354	1001	2163	2936	3028	1407	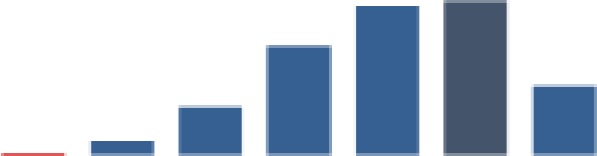
D	Total number of HIV-positive lactating mothers enrolled in the program (all lactating mothers who are in ART program)	802	1403	1727	2574	3265	3383	1658	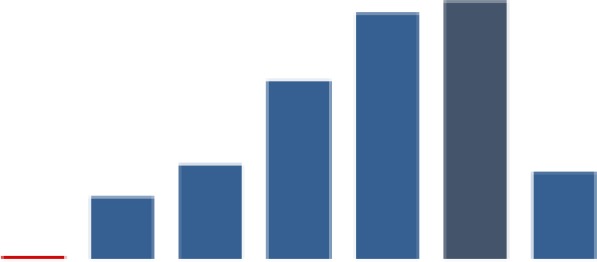
	6-month change in indicator		11.8	32.7	26.1	5.9	(0.4)	(4.6)	
	χ^2^ test		^e^	^e^	^e^	^e^	^c^	^e^	
	Number of facilities reporting	15	26	29	29	30	30	19	
**3**	**Percentage of HIV-positive pregnant and lactating women on ART in the month**	**10.9**	**89.8**	**78.1**	**86.9**	**88.1**	**86.2**	**92.4**	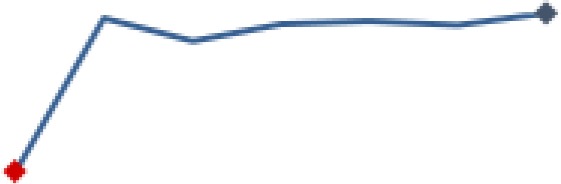
N	Number of HIV-positive pregnant and lactating women actively on ART	25	521	1660	3544	4102	4587	2078	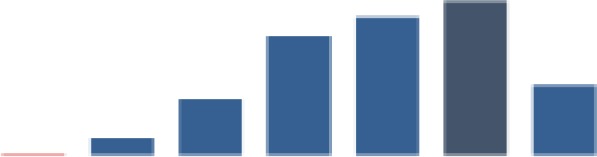
D	Total number of HIV-positive pregnant and lactating women during the reporting period (all HIV-positive pregnant and lactating women who are supposed to get ARV in that month) (cumulative)	229	580	2125	4076	4654	5322	2250	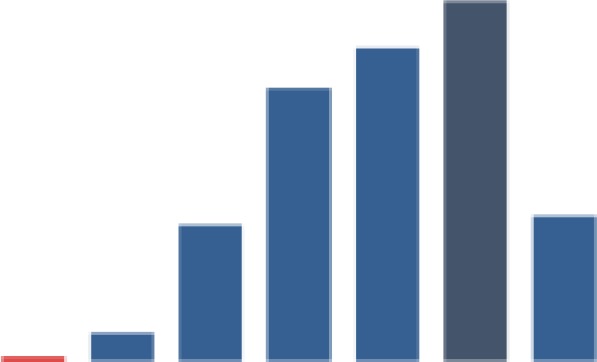
	6-month change in indicator		78.9	(11.7)	8.8	1.2	(1.9)	6.2	
	χ^2^ test		^e^	^e^	^e^	^c^	^e^	^e^	
	Number of facilities reporting	22	26	30	30	30	30	19	
**4**	**Percentage of exposed infants tested for HIV through PCR and receive results**	**69.5**	**20.2**	**62.8**	**86.7**	**79.8**	**81.9**	**96.9**	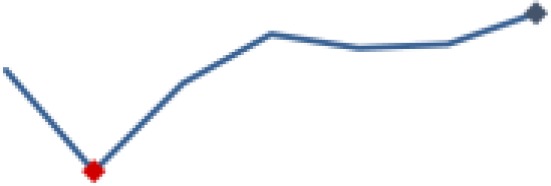
N	Number of results given to parents/guardians within 4 weeks after they reached the facility	82	18	103	183	166	122	62	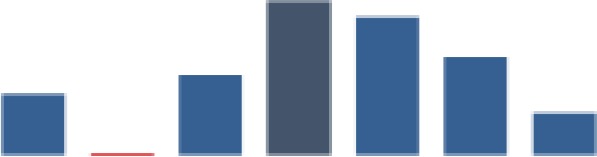
D	Total number of results received at the facility in the reporting month	118	89	164	211	208	149	64	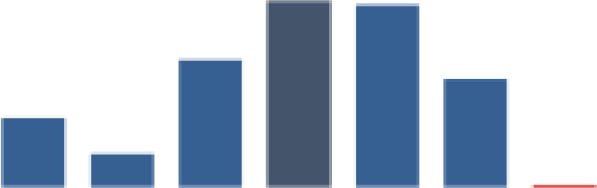
	6-month change in indicator		(49.3)	42.6	23.9	(6.9)	2.1	15.0	
	χ^2^ test		^e^	^e^	^e^	^e^	^c^	^d^	
	Number of facilities reporting	16	23	29	28	30	30	13	
**5**	**Percentage of HIV-positive pregnant women and postnatal women who attend RCH and are counseled on nutrition**	**61.3**	**13.8**	**74.5**	**70.8**	**91.2**	**98.2**	**94.2**	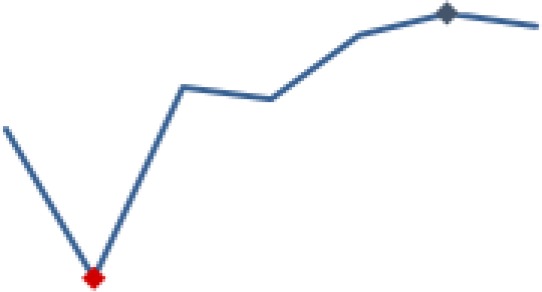
N	Number of HIV-positive pregnant women and postnatal women who attend RCH and are counseled on nutrition	38	45	1332	1895	2978	4490	1853	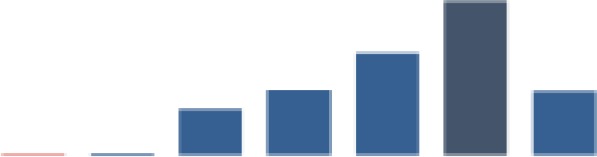
D	Total number of HIV-positive pregnant and postnatal women who are assessed for nutrition during the reporting month	62	327	1788	2676	3266	4574	1968	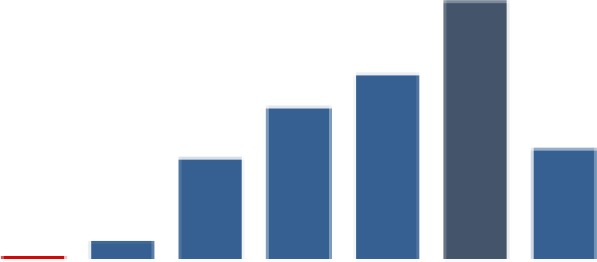
	6-month change in indicator		(47.5)	60.7	(3.7)	20.4	7.0	(4.0)	
	χ^2^ test		^e^	^e^	^d^	^e^	^e^	^e^	
	Number of facilities reporting	9	20	29	27	29	30	16	

Abbreviations: ART, antiretroviral therapy; ARV, antiretroviral; PCR, polymerase chain reaction; RCH, reproductive and child health.

^a^Negative gains or decreases are shown in red.

^b^Each period compares the change in patients receiving care at 6-month intervals.

^c^ns *P* > .05.

^d^ *P* ≤ .01.

^e^ *P* ≤ .001.

Improvement in indicator 2 was faster than indicator 1 in the first year. However, once indicator 2 surpassed the median of 82.9%, it only fell once. This could be attributed more to reporting issues rather than programmatic concerns with the facilities in Nzega, which was also the highest performing district among the 3. The mean for this measure was higher than the mean for indicator 1 at 65.4% (SD = 29.5) and ranged from 7% to 92.1%. Like indicator 1, the 6-month improvement gains were highly significant for the first 4 periods, especially in the first 3 periods: period 1 (11.8%, *P* ≤ .001), period 2 (32.7%, *P* ≤ .001), and period 3 (26.1%, *P* ≤ .001). By period 4, the improvement (5.9%, *P* ≤.001) was comparatively lower than the first 3 periods; a slight downward trend occurred in period 4 (0.4%) that significantly decreased further in period 6 (4.6%, *P* ≤ .001).

#### Uptake of ART among pregnant women and lactating mothers

**Figure 6.**
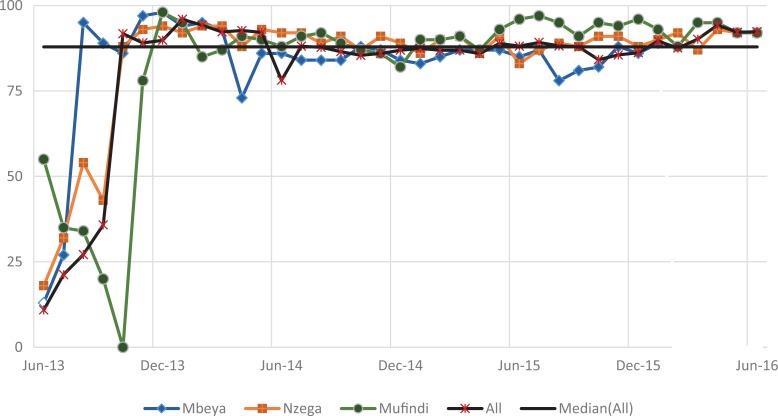
Pregnant and lactating women on ARTs in the month (June 2013 to June 2016). ART indicates antiretroviral therapy.

Figure 6 shows the trend for the pregnant and lactating women on ART each month (indicator 3). Improvement was rapid as shown by the steep slope for all 3 districts in the first year. The aggregate indicators stabilized somewhere around the median of 87.9% in October 2013 (91.8%). From then on, the indicator was either slightly below or above the median. All 3 districts followed a similar trend, with minimal difference in the indicators over time. There was a significantly high improvement gain (78.9%, *P* ≤ .001) from baseline (10.9%). A significant decrease (11.7%, *P* ≤ .001) followed in period 2. However, the indicator was able to move up again to 86.9%, with a relatively small increase in the following period. The significant decrease (1.9%, *P* ≤ .001) in period 5 was followed by a higher significant improvement in period 6 (6.2%, *P* ≤ .001), with the indicator reaching 92.4% by end of the reporting period.

#### Identification of HIV-positive infants through testing

**Figure 7.**
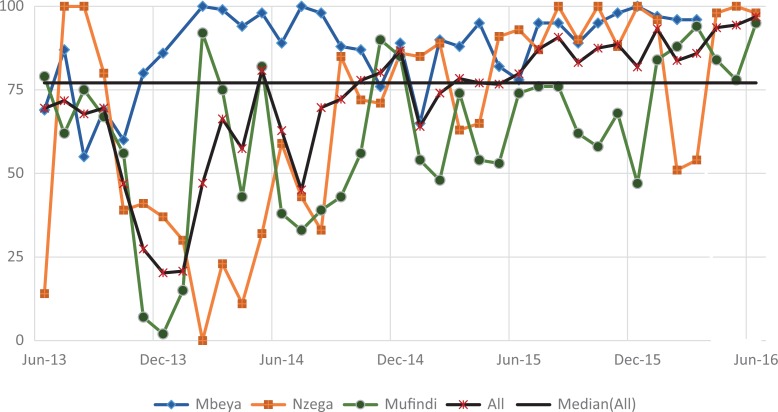
Exposed infants tested for HIV through DNA PCR and receiving results (June 2013 to June 2016). PCR indicates polymerase chain reaction.

Figure 7 shows indicator 4, which was the percentage of HEI tested for HIV with documented results. There is variation across the 3 districts for this indicator. Mbeya was generally higher than Mufindi and Nzega, which interchanged places for the lowest indicators over time. The aggregate measure for the 3 districts moved above the median (77.1%) after 2 years from baseline. The mean indicator was 71.8% (SD = 19.7). An initial high decrease (49.3%, *P* ≤ .001) occurred in period 1, followed by a similarly high and significant gain (42.6%, *P* ≤ .001) in period 2. A further significant increase (23.9%, *P* ≤ .001) occurred in period 2. By period 3, the indicator fell to 79.8% with a significant decrease (6.9%, *P* ≤ .001), followed by a slight gain of 2.1% in period 5, and then a significant increase (15%, *P* ≤ .01) to 96.9% at the end of the reporting period.

#### Counseling HIV-positive women on nutrition

**Figure 8.**
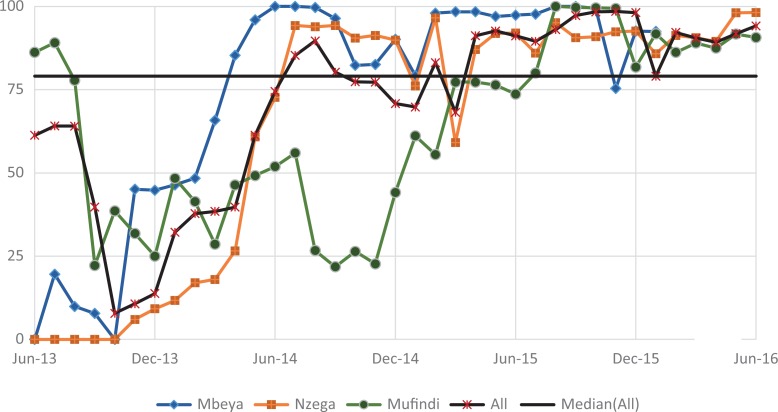
HIV-positive pregnant women and those in postnatal care who attend RCH and are counseled on nutrition (June 2013 to June 2016). RCH indicates reproductive and child health.

Figure 8 shows indicator 5, the percentage of pregnant women and those in postnatal care who benefited from the NACS package. Like indicator 4, there was variation across the 3 districts, with Mbeya consistently reporting higher values from baseline but which fell below both other regions in the past 6 months of the reporting period. Although Nzega started out with the lowest performance, it achieved the second highest performance after Mbeya by June 2014. The fluctuations in this indicator are marked by the 3 periods of significant gains and 3 periods of significant decrease. The highest increase occurred in period 2 (60.7, *P* ≤ .001), and this surpassed the initially high and significant decrease (47.5%, *P* ≤ .001) in period 1.

There was a further decrease (3.7%, *P* ≤ .01) in period 3, followed by consecutive gains in period 4 (20.4%, *P* ≤ .001) and period 5 (7.0%, *P* ≤ .001). Although there was a slight but significant decrease (4%, *P* ≤ .001)) in period 6, the indicator ended at a high of 94.5%.

## Discussion

From the beginning of PHFS implementation in Tanzania, the process was collaborative, with leadership by the MOHCDGEC and technical oversight for QI from the USAID–funded ASSIST project. The collaborative nature of the partnership was critical; it ensured all members were in alignment and involved with setting realistic goals that in turn facilitated adherence to a common protocol and evaluative framework. Moreover, district ownership for the program enabled the CHMT to improve its capacity to monitor the PMTCT program.

All indicators improved to above 80% by the end of the reporting period. Retention to care is critical to assuring treatment for HIV-positive mothers and identifying exposed infants. Indicators 1 and 2 increased at almost a similar rate over time. However, there was more variation in indicator 1, which looked at all 4 PNC visits. Indicator 1 considers all 4 visits for 1 patient, and fluctuating attendance will impact the improvement, whereas indicator 2 focuses on a smaller sample of HIV-positive mothers, during one visit in a month. Improving retention in care can directly improve the uptake of ARV drugs among HIV-positive pregnant women. This indicator remained high and consistent over the 3-year period.

A notable accomplishment of this program was how community partners’ visibility and role as stakeholders increased due to their engagement in the NSC. Community actors were effective in tracking pregnant and lactating women; community volunteers (ie, HBC workers, expert PLHIV patients, and mother-to-mother groups) helped increase attendance at ANC and RCH services. This improved patient retention (as shown in indicators 1 and 2), which was critical for ensuring ARV drug uptake and adherence.

The PHFS emphasized integrating a nutritional component. Indicator 5 measured counseling of HIV-positive pregnant women and those from the PNC who attended the RCH monthly. It is possible that irregular attendance at PNC affected the success at counseling HIV-positive pregnant women and those from the PNC who attended the RCH monthly. Health workers were assigned to counsel the mothers, but some mothers skipped the counseling or found that there was lack of trained staff at the health facility during their visit. The community workers were instrumental in supporting health facility staff by promoting healthy IYCF practices and assisting women with strategies to ensure optimal nutrition for themselves and their infants.

Another important aspect of the PHFS initiative was the investments made in building QI capacity among health staff and the collaborative approach to QI, which included consistent site-, district-, and national-level learning and mentoring opportunities for sharing knowledge. The use of QI resulted in the identification of changes that supported improvement in various areas over the 3-year implementation period.

## Strengths and Limitations

Quality improvement is a data-driven process; at the start of implementation, team had uncovered inadequacies with documentation at the health facilities. Service delivery and patient data were incomplete, inaccurate, or, in some cases, entirely unavailable. Teams did not possess basic information about who they were seeing, who did not access care, and which services were not being delivered to mothers and their infants. Without these data, they did now know what to improve and where to start. The NSC made improving data quality a priority by identifying the relevant issues and working to address them. One such issue was the lack of time available for already overworked health workers to complete facility registers and patient records. This challenge was addressed by involving community volunteers to assist health workers with documentation.

Not all issues identified through QI could be easily addressed by teams due to circumstances beyond their scope or control. For example, ARV stock shortages have been regularly reported by health facilities in Tanzania.^17,18^ This issue requires a concrete solution that spans the entire heath system, as lack of ARV drug puts the PLHIV at risk and could lead to a larger problem of drug resistance.^19^

Communicating the HEI polymerase chain reaction (PCR) test results to parents/guardians showed variation across the 3 districts, but improvement was recorded after introducing the change of communicating results by mobile phone to parents/guardians. A study in northern Tanzania also documented a shorter turnaround resulting from transmitting PCR results by mobile phone from laboratory to health-care workers as part of part of additional changes to the testing process.^20^ Yet, without an active network and adequate financial resources, communication of results to the parent or guardian was not always carried out. There were also geographic challenges. In Nzega and Mufindi, the zonal laboratories that conduct the PCR tests are approximately 600 km from the sites. For example, Nzega District is about 500 km from the Bugando laboratory, and some health facilities are about 90 km from Nzega town. Additionally, there were some issues with availability of reagents at the laboratories toward the end of 2015.

A common issue during PHFS implementation was that sites were understaffed. This hampered efficient service delivery and documentation. Additionally, staff turnover meant that new staff had to be re-trained on QI processes during implementation. Another major challenge to the nutritional component was the lack of therapeutic foods, which were usually out of stock and reflected poor logistical support at the sites.

The study design did not employ control sites; therefore, secular trends cannot be ruled out, and results are not generalizable across the country. However, the Tanzanian PHFS experience serves as an example of how QI can be applied successfully in a resource-poor environment.

## Conclusion and Recommendations

Although the PHFS initiative primarily focused on improving the continuum of care for HIV-positive mothers and their babies, it applied a holistic approach that was geared toward all mothers and infants, regardless of HIV status. The PHFS experience can inform ongoing efforts to reduce MTCT rates in Tanzania through the use of the change package compiled during the QI process.^16^ To reach and sustain improvement across facilities in the country, QI must be included in the comprehensive council health plans for sustainability at the district level. National, regional, and district health and education leadership must explore ways to integrate QI content into existing health-care workers’ training and preservice education systems. It is evident that Tanzania continues to experience persistent health system issues, such as shortages in human resources, ARV stockouts, and poor access to laboratories and equipment. The NSC—through the PHFS process—was able to systematically identify and document the challenges facing them and made significant inroads to strengthen the health system using QI. These collaborative efforts have paved a clearer way forward for continuing to address these issues and making even more gains toward eradicating MTCT in Tanzania.
